# Large current modulation and tunneling magnetoresistance change by a side-gate electric field in a GaMnAs-based vertical spin metal-oxide-semiconductor field-effect transistor

**DOI:** 10.1038/s41598-018-24958-z

**Published:** 2018-05-08

**Authors:** Toshiki Kanaki, Hiroki Yamasaki, Tomohiro Koyama, Daichi Chiba, Shinobu Ohya, Masaaki Tanaka

**Affiliations:** 1 Department of Electrical Engineering and Information Systems, The University of Tokyo, 7-3-1 Hongo, Bunkyoku, Tokyo, 113-8656 Japan; 2 Department of Applied Physics, The University of Tokyo, 7-3-1 Hongo, Bunkyoku, Tokyo, 113-8656 Japan; 3 Center for Spintronics Research Network (CSRN), Graduate School of Engineering, The University of Tokyo, 7-3-1 Hongo, Bunkyo-ku, Tokyo, 113-8656 Japan; 4 Institute of Engineering Innovation, Graduate School of Engineering, The University of Tokyo, 7-3-1 Hongo, Bunkyo-ku, Tokyo, 113-8656 Japan

## Abstract

A vertical spin metal-oxide-semiconductor field-effect transistor (spin MOSFET) is a promising low-power device for the post scaling era. Here, using a ferromagnetic-semiconductor GaMnAs-based vertical spin MOSFET with a GaAs channel layer, we demonstrate a large drain-source current *I*_DS_ modulation by a gate-source voltage *V*_GS_ with a modulation ratio up to 130%, which is the largest value that has ever been reported for vertical spin field-effect transistors thus far. We find that the electric field effect on *indirect* tunneling via defect states in the GaAs channel layer is responsible for the large *I*_DS_ modulation. This device shows a tunneling magnetoresistance (TMR) ratio up to ~7%, which is larger than that of the planar-type spin MOSFETs, indicating that *I*_DS_ can be controlled by the magnetization configuration. Furthermore, we find that the TMR ratio can be modulated by *V*_GS_. This result mainly originates from the electric field modulation of the magnetic anisotropy of the GaMnAs ferromagnetic electrodes as well as the potential modulation of the nonmagnetic semiconductor GaAs channel layer. Our findings provide important progress towards high-performance vertical spin MOSFETs.

## Introduction

Reducing the power consumption in integrated circuits is an important issue that we have to tackle in the 21st century. Making volatile components non-volatile is one of the most promising approaches to this issue; various non-volatile technologies, such as reconfigurable logic circuits^1^, non-volatile power gating^2^ and magnetic random access memory, are applicable to low-power-consumption electronics. A spin metal-oxide-semiconductor field-effect transistor (spin MOSFET)^3,4^, in which source and drain electrodes are ferromagnetic materials, is a key component for realizing those applications because of the several advantages inherent in spin MOSFETs: The most important advantage of spin MOSFETs over other spintronics devices such as all-spin-logic devices^5,6^ is the high amplification capability, which is crucially important for restoring propagating signals between transistors, and thus indispensable for the application to large scale integrated circuits. Also, spin MOSFETs are compatible with existing well-matured semiconductor technologies, which makes integration with semiconductor devices very easy. Spin MOSFETs can have the same advantages of scaling circuits as those of general transistors, which is very important for miniaturization of the device from the view point of energy consumption. For practical operation of spin MOSFETs, both large current modulation by applying a gate electric field and large magnetoresistance (MR) by magnetization reversal are required. At present, lateral and vertical types of spin MOSFETs have been proposed. The lateral spin MOSFETs, in which a current flows parallel to the substrate plane and is controlled by a gate electric field applied from the top of the channel, have a large current modulation capability (5 × 10^6^%^7^, 400%^8^ and 10^7^%^9^); however, the problem is the small MR ratio (0.1%^7^, 0.005%^8^ and 0.027%^9^). Meanwhile, epitaxial magnetic heterostructures were grown using various ferromagnets and semiconductors and some magnetotransport properties were studied^10–15^. Thus, a *vertical* spin field-effect transistor (FET), in which a current flows perpendicular to the film plane and is controlled by a gate voltage applied from the *side surface* of the channel, is promising for large MR. Previously, we reported ferromagnetic-semiconductor GaMnAs-based vertical spin FETs that exhibit large MR ratios (60%^16^ and 5%^17^). A GaMnAs-based heterostructure is one of the most ideal material systems, because we can obtain high-quality single-crystalline GaMnAs/nonmagnetic semiconductor (GaAs)/GaMnAs trilayers and thus can suppress spin relaxation at the interfaces^18–21^. However, in those vertical spin FETs, the current modulation ratio by the gate voltage was small (0.5%^16^ and 20%^17^). Thus, vertical spin FETs with a large current modulation are strongly required. In addition, to further improve the performance of the vertical spin FETs, we need more profound understanding of the gate electric field effect on the spin-dependent transport.

In vertical spin FETs, as shown by our electric field simulations later, the gate electric field influences the electric potential profile only within 10 nm from the side surfaces of the intermediate channel layer, which limits the current modulation. Thus, the lateral size of vertical FETs should be decreased as much as possible for obtaining high-performance vertical spin FETs. In this study, to enhance the current modulation and to understand the electric field effect on the spin-dependent transport, we reduced the lateral size (=width of the mesas as explained later) of the GaMnAs-based vertical spin MOSFET to ~500 nm. We have successfully obtained a large current modulation by the gate electric field with a modulation ratio up to 130%, which is the largest value that has been ever reported for vertical spin FETs^16,17^. Furthermore, using the electric field simulations, we find that *indirect* tunneling mainly contributes to the observed large current modulation. These new findings are important steps to further improve the performance of the vertical spin FETs.

## Results

### Samples

**Figure 1:**
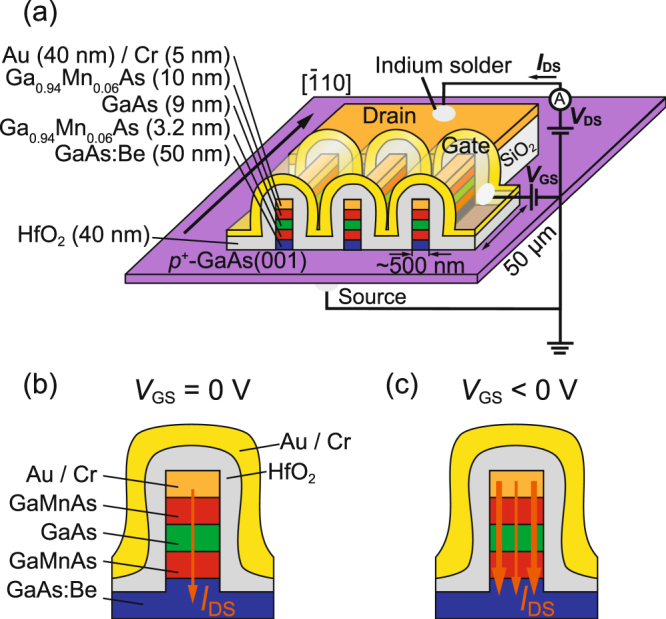
(**a**) Schematic illustration of the vertical spin MOSFET investigated in this study. The backside of the substrate is the source electrode, the comb shaped Au/Cr layer is the drain electrode and the Au/Cr layer above the HfO_2_ layer is the gate electrode. (**b**,**c**) Schematic device operation of our vertical spin MOSFET when a gate voltage *V*_GS_ is not applied (**b**) and when a negative gate voltage is applied (**c**). The orange arrows represent a drain-source current *I*_DS_.

Our vertical spin MOSFET has a thin GaAs channel (9 nm) and ferromagnetic-semiconductor GaMnAs source and drain electrodes [Fig. 1(a)] (See the Methods section). To increase the current modulation, we reduced the width of the mesas down to ~500 nm. As a gate insulator, a 40-nm-thick HfO_2_ film was used since it has a large relative permittivity, which also contributes to the increase of the current modulation. In this device, tunneling of holes occurs between the source and drain, because GaAs is a potential barrier with a height of ~100 meV for holes in the GaMnAs layers^22,23^, as shown in Fig. 1(b). When the gate-source voltage *V*_GS_ < 0 V (*V*_GS_ ≥ 0 V), the tunneling current flowing at the side surfaces of the mesas is increased (decreased), as shown in Fig. 1(c).

### MOSFET operation and its analyses

**Figure 2:**
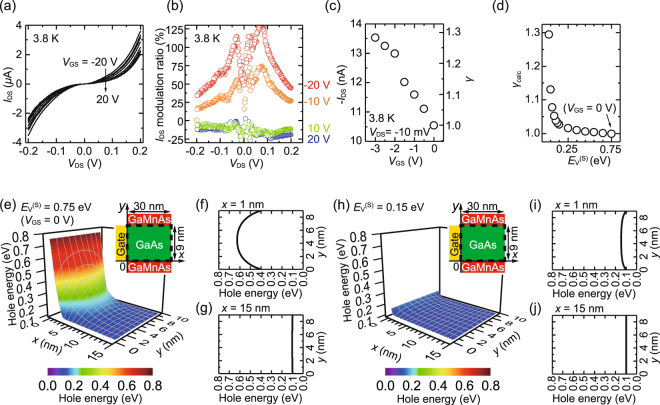
(**a**) Drain-source current *I*_DS_ as a function of the drain-source voltage *V*_DS_ with the gate-source voltage *V*_GS_ ranging from −20 V to 20 V with a step of 5 V at 3.8 K. (**b**) *I*_DS_ modulation ratio as a function of *V*_DS_ with various *V*_GS_ at 3.8 K. (**c**) Drain-source current (−*I*_DS_) (left axis) and the *I*_DS_ value normalized at *V*_GS_ = 0 V (*γ*) (right axis) as a function of *V*_GS_ with *V*_DS_ = −10 mV at 3.8 K. (**d**) Calculated *I*_DS_ normalized by the one at *V*_GS_ = 0 V (*γ*_calc_) as a function of *E*_V_^(S)^. (**e**,**h**) Calculated valence band top energy *E*_V_ with respect to the Fermi level when *E*_V_^(S)^ = 0.75 eV (**e**) and *E*_V_^(S)^ = 0.15 eV (**h**). Here, the Fermi level corresponds to 0 eV. The vertical axis expresses the hole energy. The inset in (**e**) and (**h**) shows the structure used in our calculation. Here, the *x* axis represents the distance from the side surface of the mesa and the *y* axis denotes the distance from the interface between the bottom GaMnAs layer and the intermediate GaAs layer. The calculation was performed in the region surrounded by the dashed line. In (**e**,**h**), only the region of 0 nm ≤ *x* ≤ 15 nm is shown because it is sufficient to see how the gate electric field influences the valence band maximum energy in the GaAs layer. (**f**,**g**) *E*_V_ *vs*. *y* at *x* = 1 nm (**f**) and 15 nm (**g**) when *E*_V_^(S)^ = 0.75 eV. (**i**,**j**) *E*_V_ *vs*. *y* at *x* = 1 nm (**i**) and 15 nm (**j**) when *E*_V_^(S)^ = 0.15 eV.

To investigate the MOSFET characteristics of this device, we measured the drain-source current *I*_DS_ as a function of the drain-source voltage *V*_DS_ for various *V*_GS_ [Fig. 2(a)]. Nonlinear *I*_DS_–*V*_DS_ characteristics were observed for each *V*_GS_ (black curves), indicating that tunneling transport is dominant. Furthermore, *I*_DS_ was largely controlled by *V*_GS_. We note that the gate leakage current and electric field effect on parasitic resistances (the resistances of the top/bottom GaMnAs layers, GaAs:Be layer and Au/Cr electrodes), which may induce unintended modulation of *I*_DS_, were negligibly small (see Supplementary Note 1). When *V*_GS_ = 20 V, the *I*_DS_ modulation ratio by *V*_GS_, which is defined by [*I*_DS_(*V*_GS_) – *I*_DS_(*V*_GS_ = 0 V)]/*I*_DS_(*V*_GS_ = 0 V) × 100 (%), is around −20% [see the blue points in Fig. 2(b)]. On the other hand, when *V*_GS_ = −20 V, it reached ~130% [see the red points in Fig. 2(b)]. This *I*_DS_ modulation ratio (~130%) is the largest among the values reported for vertical spin FETs thus far^16,17^.

To understand the modulation of the band alignment in detail, we measured *I*_DS_ as a function of *V*_GS_ at *V*_DS_ = −10 mV [Fig. 2(c)]. *I*_DS_ normalized at *V*_GS_ = 0 V (*γ*) was changed from 1 to 1.28 when *V*_GS_ was changed from 0 V to −3 V [see the right axis in Fig. 2(c)], meaning that *I*_DS_ was increased by 28% when *V*_GS_ was changed from 0 V to −3 V. As shown below, this large modulation of *I*_DS_ cannot be understood by the electric field effect on *direct* tunneling. To obtain the potential distribution and to calculate *I*_DS_ normalized at *V*_GS_ = 0 V (*γ*_calc_), we performed electric field simulation varying the electric potential at the side surface of the mesa and investigated the effect of the side-gate electric field [Fig. 2(e,h)] (see Supplementary Note 2). Here, we define *E*_V_^(S)^ as the valence band top energy *E*_V_ at the side surface (interface between the side-gate electrode and the GaAs channel) with respect to the Fermi level *E*_F_ in terms of hole energy. The potential profile of *E*_V_ when *E*_V_^(S)^ = 0.75 eV is shown in Fig. 2(e), which corresponds to the case of *V*_GS_ = 0 V, because *E*_F_ at the side surface of the GaAs channel is pinned at the middle of the band gap^24^. With decreasing *E*_V_^(S)^ from 0.75 eV, the electric potential near the side surface of the mesa is decreased [Fig. 2(f,i)], whereas the electric potential in the inner region of the mesa (10 nm ≤ *x*) is not influenced [Fig. 2(g,j)]. As shown in Fig. 2(d), *γ*_calc_ remains almost unchanged between Fig. 2(e) (*γ*_calc_ = 1 when *E*_V_^(S)^ = 0.75 eV) and (h) (*γ*_calc_ = 1.028 when *E*_V_^(S)^ = 0.15 eV) because GaAs is a potential barrier for holes in both cases. On the other hand, when *E*_V_^(S)^ < 0.15 eV, because *E*_V_ of the GaAs channel at the side surface becomes lower than *E*_V_ inside the mesa, *γ*_calc_ starts to increase with decreasing *E*_V_^(S)^ [Fig. 2(d)]. This feature is different from the experimental data shown in Fig. 2(c); when *V*_GS_ is changed from 0 V to −10 V, *γ* starts to increase at *V*_GS_ = 0 V. We can see the significant difference in the curve shapes of Fig. 2(c) and Fig. 2(d). This analysis indicates that the electric field effect on the *direct* tunneling current *cannot* reproduce the experimental *I*_DS_–*V*_GS_ characteristic. Instead, the main origin of the large modulation ratio observed in our device is the electric field effect on the *indirect* tunneling current^25^.

The indirect tunneling current is probably caused by a large amount of Mn atoms (~10^18^ cm^−3^), which are diffused to the intermediate GaAs layer from the top and bottom GaMnAs layers and form defect states in the band gap of GaAs. Furthermore, GaAs grown at low temperature (200 °C) is known to have a large amount of arsenic antisite defects (10^18^–10^19^ cm^−3^)^26,27^. In fact, the *I*_DS_–*V*_DS_ characteristics of our device show a strong temperature dependence (see Supplementary Note 3), which indicates that indirect tunneling via defect states takes place. (If *I*_DS_ were dominated only by the direct tunneling current, no temperature dependence would be observed.) Therefore, the electric field effect on indirect tunneling via defect states is the most probable origin for the large *I*_DS_ modulation ratio. To enhance the *I*_DS_ modulation ratio, we have to solve the Fermi level pinning problem at the surface of GaAs, i.e., because the Fermi level is severely pinned at the middle of the band gap at the surface of GaAs, it is very difficult to move the valence band maximum toward the Fermi level of the GaMnAs layers by the application of *V*_GS_. The Fermi level pinning in GaAs is thought to originate from defect states formed at the interface between GaAs and gate insulators. Thus, reducing the interface state density between GaAs and gate insulators is a key to enhancing the current modulation ratio by *V*_GS_.

### Tunneling magnetoresistance and its change by *V*_GS_

**Figure 3:**
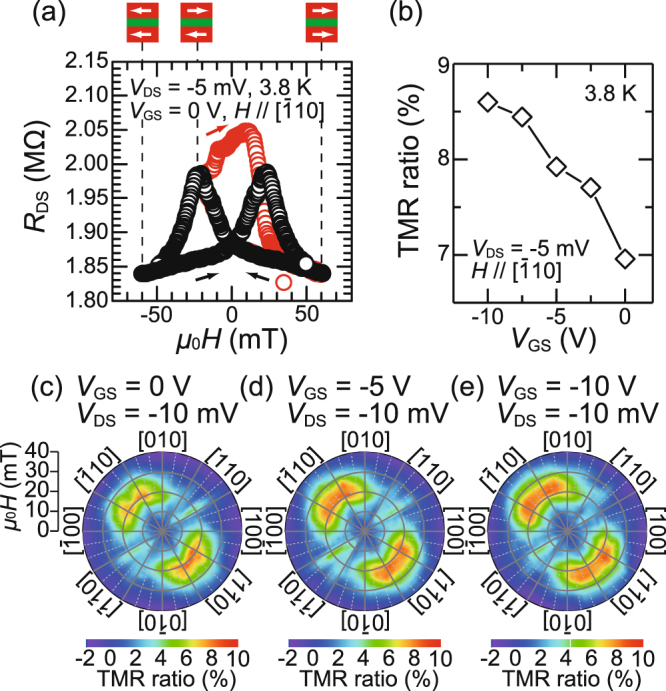
(**a**) Drain-source resistance *R*_DS_ as a function of the in-plane external magnetic field *μ*_0_*H* applied along the [$\bar{1}$10] direction at 3.8 K. Here, the drain-source voltage *V*_DS_ was −5 mV and the gate-source voltage *V*_GS_ was 0 V. The black circles correspond to the major loop and the red circles correspond to the minor loop. The black (red) arrows are the sweep directions in the major (minor) loop. The magnetization states in the major loop are indicated by the white arrows above the graph. (**b**) TMR ratio as a function of the gate-source voltage *V*_GS_ at 3.8 K. Here, the drain-source voltage *V*_DS_ was fixed at −5 mV and the external magnetic field *H* was applied along the [$\bar{1}$10] direction. The TMR ratio is the maximum value obtained in the major loop at each *V*_GS_. (**c**–**e**) Magnetic-field-direction dependences of the TMR ratios at *V*_DS_ = −10 mV with *V*_GS_ = 0 V (**c**), -5 V (**d**) and −10 V (**e**).

To investigate the spin-dependent transport of our device, we measured the drain-source resistance *R*_DS_ as a function of *μ*_0_*H* applied along the [$\bar{1}$10] direction in the film plane with *V*_DS_ = −5 mV and *V*_GS_ = 0 V [Fig. 3(a)]. Here, *R*_DS_ is defined by *V*_DS_/*I*_DS,_ *μ*_0_ is the permeability of a vacuum and *H* is an in-plane external magnetic field. In the major loop (black circles), clear tunnel magnetoresistance (TMR) was observed, indicating that *I*_DS_ can be controlled by the magnetization configuration. The TMR ratio, which is defined by [*R*_DS_(*μ*_0_*H*) – *R*_DS_(*μ*_0_*H = *0 mT)]/*R*_DS_(*μ*_0_*H* = 0 mT) × 100 (%), reached ~7% at *μ*_0_*H* = 20 mT, where *R*_DS_(*μ*_0_*H*) is the drain-source resistance at *H* in the major loop. This value is more than 70 times larger than the MR ratios obtained in the lateral spin MOSFETs^7–9^. In the major loop, the negative MR was observed at *μ*_0_*H* < 0 mT (*μ*_0_*H* > 0 mT) when *μ*_0_*H* was swept from positive to negative (negative to positive) probably because of a multi-domain structure in the top and bottom GaMnAs layers. We also observed a clear minor loop (red circles), indicating that the antiparallel magnetization configuration is stable even at *μ*_0_*H* = 0 mT. (In the minor loop, *R*_DS_ increases with increasing *μ*_0_*H* from −20 mT to 10 mT, probably because the magnetizations of the top and bottom GaMnAs layers are not completely antiparallel at the peak of *R*_DS_ (at *μ*_0_*H* = −20 mT in the major loop) and they become close to the perfect antiparallel configuration with increasing *μ*_0_*H* to 10 mT in the minor loop.)

To investigate the influence of *V*_GS_ on the spin-dependent transport, we measured the *V*_GS_ dependence of the TMR ratio [Fig. 3(b)]. Here, the TMR ratio corresponds to the maximum value obtained in the major loop at each *V*_GS_. The TMR ratio tends to increase as *V*_GS_ is changed from 0 V to −10 V. In our device, the gate electric field can modulate the electronic states of the top/bottom GaMnAs layers as well as those of the intermediate GaAs layer, both of which can modulate TMR. Applying *V*_GS_ causes the change in the hole density of the GaMnAs layers near the side surfaces of the mesas, which can change the spin polarization and magnetic anisotropy^17^. To understand the modulation of the magnetic anisotropy by *V*_GS_ in our device, we measured TMR applying *H* in various in-plane directions with an angle *θ* with respect to the [100] axis in the counterclockwise rotation when *V*_GS_ = 0, −5 and −10 V [Fig. 3(c–e)] (see Measurements section). The observed TMR ratios showed dominant uniaxial anisotropy along the [$\bar{1}$10] direction in addition to biaxial anisotropy along the <100> directions for any *V*_GS_ [see the four red peaks in Fig. 3(c–e)]. With changing *V*_GS_ from 0 V to −10 V, the easy axes of our device were slightly rotated toward the [010] direction (the red-colored region is extended toward the [010] direction). In spintronics devices, changing the magnetization directions is necessary to write information, which accompanies the power consumption. The controllability of the magnetic anisotropy by the gate voltage is more advantageous in spintronics devices than other methods such as optical control of the magnetic properties^28^ because we can expect the reduction of the power consumption required for writing information, since the application of the gate voltage does not accompany joule heating. Furthermore, the coercive force of the top GaMnAs layer, which has a larger coercivity than the bottom GaMnAs layer, increases as *V*_GS_ is changed from 0 V to −10 V (the red-colored region slightly expands outward). These results indicate that the magnetic anisotropy constants are modulated by applying negative *V*_GS_, which can also contribute to the modulation of the TMR ratio. In addition, *V*_GS_ modifies the valence band maximum energy in the GaAs layer. As we discussed in the previous section, the modulation of the indirect tunneling current via defect states is the most probable mechanism for the obtained large modulation of *I*_DS_. The TMR induced by indirect tunneling via defect states depends on many factors such as energy levels of defect states, band width of defect states, life time of carries and so on. The modulation of the electric potential can influence the indirect tunneling and thus TMR. Therefore, the electric field effect both on the top/bottom GaMnAs layers and on the intermediate GaAs layer contributes to the modulation of the observed TMR ratio. Although only the side surfaces of the mesas are influenced by *V*_GS_, the MR and the magnetic anisotropy of our device are clearly modulated. This phenomenon can be understood as follows. When negative *V*_GS_ is applied, the current flowing at the side surface of the mesas is increased and becomes dominant in *I*_DS_. When negative *V*_GS_ is applied, the magnetic properties of the side surface of the top and bottom GaMnAs layers are also modulated. These facts indicate that the modulation of the magnetic properties of the top and bottom GaMnAs layers, which takes place only at the edge regions of the mesas, is preferentially reflected to the measured spin-dependent transport properties in our device. Therefore, the MR and the magnetic anisotropy of our device are modulated although only the edge regions of the mesas are influenced by *V*_GS_.

Surprisingly, the *V*_GS_ dependence of the TMR ratio shown in Fig. 3(b) is completely opposite to the one obtained in our previous study, *i*.*e*. the TMR ratio *decreased* as negative *V*_GS_ is applied in our previous study^16^. This may be caused by the difference of the easy axes between the present device and the previous one (the biaxial anisotropy was dominant in our previous work) or by the different direction of an external magnetic field (along the direction with an angle 10-degree from the [100] direction toward the [1 $\bar{1}$ 0] in our previous work).

## Summary

In summary, we have investigated the electric field effect on the spin-dependent transport properties in a GaMnAs-based vertical spin MOSFET. We obtained a large current modulation ratio up to 130%, which is the largest value that has ever been reported thus far for the vertical spin FETs^16,17^. By comparing the experimental data with the calculated results, we concluded that this large *I*_DS_ modulation does not originate from the modulation of direct tunneling between the source and the drain but from the modulation of the indirect tunneling current via defect states in the intermediate GaAs layer. The TMR ratio tends to increase as negative *V*_GS_ is applied, which is attributed to the electric field effect both on the top/bottom GaMnAs layers and on the intermediate GaAs layer. These results provide an important insight into the device physics for realizing high-performance vertical spin MOSFETs.

## Methods

### Growth

The heterostructure composed of, from the top to the bottom, Ga_0.94_Mn_0.06_As (10 nm)/GaAs (9 nm)/Ga_0.94_Mn_0.06_As (3.2 nm)/GaAs:Be (50 nm, hole concentration *p* = 5 × 10^18^ cm^−3^) on a *p*^+^-GaAs (001) substrate by low-temperature molecular beam epitaxy. The growth temperatures of the top Ga_0.94_Mn_0.06_As, GaAs, bottom Ga_0.94_Mn_0.06_As and GaAs:Be layers were 195 °C, 180 °C, 200 °C and 520 °C, respectively. The Curie temperatures of the top and bottom GaMnAs layers were estimated to be ~53 K and 20–30 K, respectively. (For the details, see the Supplementary Note 4). Post-growth annealing was not carried out.

### Process

After the growth, we partially etched the grown films and buried the etched area with a 100-nm-thick SiO_2_ layer for the separation of the drain electrode and the substrate. Then, a comb-shaped Au (40 nm)/Cr (5 nm) layer, whose width of the comb teeth is ~500 nm and length of them is 50 μm, was formed by electron-beam lithography and a lift-off technique. We chemically etched the area that is not covered by the Au/Cr layer and then the magnetic tunnel junctions only beneath the comb teeth area of the Au/Cr drain electrode remained after the etching. Since the narrow-line-shaped mesas are formed by chemical wet etching, the influence of defects at the side surfaces of the GaAs layer induced by the *nanofabrication* of the mesas can be neglected. We formed a 40-nm-thick HfO_2_ film as a gate insulator using atomic layer deposition at a substrate temperature of 150 °C and deposited a gate electrode composed of Au (50 nm)/Cr (5 nm) by electron-beam deposition.

### Measurements

After the device was bonded with Au wires and indium solder, we measured the spin-dependent transport properties of our spin MOSFET with varying *V*_GS_ and *H* at 3.8 K. Here, the experimental data of our vertical spin MOSFET are collected from 20 mesas that are connected by the drain electrode in the comb-shaped chip. To measure the *θ* dependence of TMR, we applied a strong magnetic field of 1 T in the opposite direction of *θ* to align the magnetization directions and we decreased *H* to zero. Then, we started to measure *R*_DS_ while increasing *H* from zero in the direction of *θ*. The measurements were performed at every 10° step of *θ*. We note that our experimental data are reproducible.

## Electronic supplementary material

Supplementary Information
