# Supplementary material for: Large current modulation and tunneling magnetoresistance change by a side-gate electric field in a GaMnAs-based vertical spin metal-oxide-semiconductor field-effect transistor
Source: Sci Rep. 2018 May 8;8:7195. doi: 10.1038/s41598-018-24958-z (PMC5940878; doi:10.1038/s41598-018-24958-z)
Supplement: Supplementary file 1 — Supplementary Information [file 41598_2018_24958_MOESM1_ESM.pdf]

Supplementary information

Large current modulation and tunneling magnetoresistance change by a side-gate electric field in a GaMnAs-based vertical spin metal-oxide-semiconductor field-effect transistor

Toshiki Kanaki, Hiroki Yamasaki, Tomohiro Koyama, Daichi Chiba, Shinobu Ohya and Masaaki Tanaka

Supplementary Note 1. Influence of the gate leakage current and electric field effect on the parasitic resistances

The observed gate-source leakage current ranged from -400 pA to 200 pA. It is much smaller than the experimentally observed drain-source current (I_{DS}) modulation (~0.36 μA at the drain-source voltage (V_{DS}) of 80 mV and the gate-source voltage (V_{GS}) of -20 V). Thus, the gate leakage current is negligibly small. The influence of the gate electric field on the parasitic resistances is also negligibly small. The resistances of the top GaMnAs layer, bottom GaMnAs layer, GaAs:Be layer and Au/Cr layers are estimated to be 1.6 mΩ, 0.5 mΩ, 20 mΩ and 10 μΩ, respectively. Here, the resistances of the top and bottom GaMnAs layers, GaAs:Be layer and Au/Cr layers are obtained using the area of the mesas, the thickness of each layer and the resistivity of GaMnAs, GaAs:Be and Au/Cr. The resistivity of the GaMnAs, GaAs:Be and Au/Cr layers was measured to be 4 mΩ.cm, 10 mΩ.cm and 5.8 μΩ.cm, respectively, using Hall-bar structures. The modulation of resistance in the GaAs:Be layer by a gate electric field was also measured to be ~3% at 3.8 K using a Hall-bar structure with a gate electrode. Because the carrier concentration of the GaMnAs layers and Au/Cr layers are much larger than that of the GaAs:Be layer, the modulation of resistance in the top and bottom GaMnAs layers and Au/Cr layers is expected to be much less than 3%. Considering that the drain-source resistance changes from 80 kΩ (at V_{DS} = 200 mV) to 1.5 MΩ (at V_{DS} = 5 mV) at 3.8 K (see the purple curve in Fig. S2), the modulation of the parasitic resistances is (1.6 mΩ + 0.5 mΩ + 20 mΩ + 10 μΩ)×3% / 80 kΩ ~0.8×10^{-6}% at most, which is much smaller than the experimentally obtained value (~130% at V_{DS} = 80 mV and V_{GS} = -20 V). Thus, the influence of the gate leakage current and electric field effect on the parasitic resistances is negligibly small.

Supplementary Note 2. Details of the calculation of the electric potential profile and the normalized drain-source current in our vertical spin MOSFET

The device structure used in our simulation is shown in Fig. S1. To calculate the electric potential profile with various gate voltages, we solved the following two-dimensional Poisson equation.

$$\frac{\partial^2 E_{\mathrm{V}}}{\partial x^2} + \frac{\partial E_{\mathrm{V}}{}^2}{\partial y^2} = -\frac{\rho}{\epsilon}. \tag{S1}$$

Here, E_{V} is the valence band top energy of GaAs in terms of hole energy, ρ is the charge density and ε is the dielectric constant of GaAs, respectively. Because all the experimental results presented in the main text were obtained at 3.8 K, activation of donors/acceptors and thermally excited carriers can be neglected in GaAs. Thus, we set the charge density at 0 in GaAs, meaning that the right side of Equation (S1) is 0.

Considering that the potential barrier height of GaAs is ~0.1 eV for holes in the GaMnAs layers, Equation (S1) is solved under the following boundary conditions.

- At $x = 0$, $E_{\mathrm{V}} - E_{\mathrm{F}} = E_{\mathrm{V}}{}^{(\mathrm{S})}$.
- At $x = W$, $E_{\mathrm{V}} - E_{\mathrm{F}} = 0.1$ eV.[1,2]

Here, E_{F} is the Fermi level, $E_{\mathrm{V}}{}^{(\mathrm{S})}$ is the valence band top energy with respect to E_{F} at x = 0 (the interface between the side-gate electrode and GaAs), L is the channel length and W is the width of the calculated region [see Fig. S1].

As the boundary condition at the interfaces of GaMnAs/GaAs (y = 0 and L), we used the E_{V} profile obtained for GaMnAs; in the surface depletion region of GaMnAs ($0 \leq x \leq W_{\mathrm{D}}$), where W_{D} is the width of the depletion layer of GaMnAs, E_{V} has a parabolic form that satisfies $E_{\mathrm{V}} - E_{\mathrm{F}} = E_{\mathrm{V}}{}^{(\mathrm{S})}$ at $x = 0$. When $W_{\mathrm{D}} \leq x$, $E_{\mathrm{V}} - E_{\mathrm{F}}$ was approximated to be 0.1 eV, which is the same as the barrier height of GaAs for holes, in GaMnAs. Considering the above conditions, the energy profile at y = 0 and L can be expressed by the following equation.

$$E_{\mathrm{V}} - E_{\mathrm{F}} = \begin{cases} \left(E_{\mathrm{V}}^{(\mathrm{S})} - 0.1\right)\left(1 - \dfrac{x}{W_{\mathrm{D}}}\right)^2 + 0.1 & (0 \leq x \leq W_{\mathrm{D}}) \\ 0.1 & (W_{\mathrm{D}} \leq x) \end{cases}. \tag{S2}$$

The depletion layer width W_D is expressed by

$$W_D = \sqrt{\frac{2\epsilon|E_V^{(S)}|}{eN_A}}. \tag{S3}$$

Here, N_A is an acceptor concentration of GaMnAs and we set N_A at 10^{20} cm^{-3}. To introduce the effect of the gate electric field, we changed $E_V^{(S)}$. When a gate voltage is not applied, $E_V^{(S)} = E_g/2$, where E_g is the band gap of GaAs, because of the Fermi level pinning at the surface of GaAs. We set W = 30 nm, which is larger than the depletion layer width in the GaAs layer (2–15 nm) [see Fig. 2(e,h)]. The parameters used in the potential calculation are summarized in Table S1. Equation (S1) was solved using Jacobi's iterative method so that the potential difference between the present step and the previous step at all (x, y) becomes less than 10^{-9} eV. Each mesh is a rectangle with a width in the x direction (Δx) of 0.1 nm and a width in the y direction (Δy) of 0.1 nm.

The drain-source current I_{DS} at $E_V^{(S)}$ can be expressed by the following equation.

$$I_{DS} = 2\int_{W_D}^{\frac{W_{mesa}}{2}} J_{E_V^{(S)}}(x)dx \times L_{mesa} \times N_{mesa}. \tag{S4}$$

Here, W_{mesa} is the width of our mesa (500 nm), $J_{E_V^{(s)}}(x)$ is the current density at x and $E_V^{(S)}$, L_{mesa} is the length of our mesa (50 μm) and N_{mesa} is the number of the mesas (10). When the applied voltage between the source and the drain $|V|$ is much smaller than the barrier height, the carrier energy E dependence of tunneling probability can be neglected. Thus, $J_{E_V^{(s)}}(x)$ can be described by

$$\begin{aligned} J_{E_V^{(S)}}(x) &\propto \int_{-eV}^{0} D_{top}(E)D_{bot}(E+eV)T_{E_V^{(S)}}(x)\,dE \\ &= T_{E_V^{(S)}}(x) \times \int_{-eV}^{0} D_{top}(E)D_{bot}(E+eV)\,dE. \end{aligned} \tag{S5}$$

Here, D_{top}, D_{bot} and $T_{E_V^{(S)}}(x)$ are the density of states in the top GaMnAs layer, the

density of states in the bottom GaMnAs layer and the tunneling probability at x and $E_V^{(S)}$, respectively.

Using equation (S4) and (S5), we can obtain the following relationship between I_{DS} and $T_{E_V^{(S)}}(x)$.

$$I_{DS} \propto 2\int_{W_D}^{\frac{W_{mesa}}{2}} T_{E_V^{(S)}}(x)dx. \tag{S6}$$

Note that we consider that D_{top} and D_{bot} do not depend on $E_V^{(S)}$. Therefore, only $T_{E_V^{(S)}}(x)$ is dependent on $E_V^{(S)}$.

$T_{E_V^{(S)}}(x)$ can be calculated using the Wentzel-Kramers-Brilluion approximation. The right side of Equation (S6) can be calculated as follows.

$$\begin{aligned} 2\int_{W_D}^{\frac{W_{mesa}}{2}} T_{E_V^{(S)}}(x)dx \\ &= 2\int_{W_D}^{W} \exp\left(-2\int_0^L \frac{\sqrt{2m_h\left(E_{V,E_V^{(S)}}(x,y)-E_F\right)}}{\hbar}dy\right)dx \\ &+ 2\left(\frac{W_{mesa}}{2}-W\right) \\ &\times \exp\left(-2\int_0^L \frac{\sqrt{2m_h\left(E_{V,E_V^{(S)}}(x=W,y)-E_F\right)}}{\hbar}dy\right). \end{aligned} \tag{S7}$$

Here, $E_{V,E_V^{(S)}}(x,y)$ is the valence band top energy at (x, y) and $E_V^{(S)}$ and $\hbar$ is the reduced Planck constant.

Thus, the calculated I_{DS} normalized at V_{GS} = 0 V, corresponding to $E_V^{(S)} = E_g/2$, (γ_{calc}) can be described as follows.

$$\gamma_{\mathrm{calc}} = \int_{W_{\mathrm{D}}}^{\frac{W_{\mathrm{mesa}}}{2}} T_{E_{\mathrm{V}}^{(\mathrm{S})}}(x)dx \Bigg/ \int_{W_{\mathrm{D}}}^{\frac{W_{\mathrm{mesa}}}{2}} T_{E_{\mathrm{V}}^{(\mathrm{S})}=\frac{E_{\mathrm{g}}}{2}}(x)dx. \quad \text{(S8)}$$

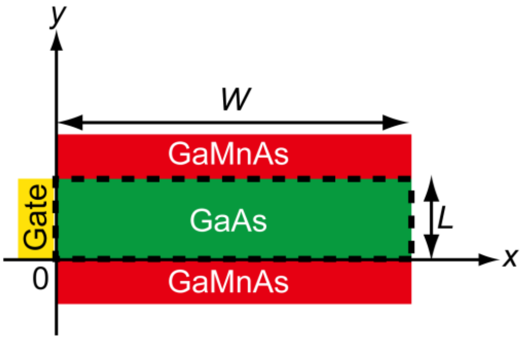

FIG. S1. Schematic illustration of the device structure used in our electric potential calculation. Here, L is the GaAs-channel length (9 nm) and W is the width of the calculated area surrounded by the dashed lines. In our calculation, W was set at 30 nm, which is large enough to see how the gate electric field influences the electric potential profiles.

TABLE S1. Parameters used in the calculation of the electric potential profiles and γ_{calc}.

Parameters (unit)	Values
L (nm)	9
W (nm)	30
Δx (nm)	0.1
Δy (nm)	0.1
m_h (kg)	$0.45m_0$ [1,2]
E_g (eV)	1.51914

Supplementary Note 3. Temperature dependence of the drain-source resistance $\boldsymbol{R}_{\mathbf{DS}}$.

We show R_{DS} *vs.* V_{DS} at various temperatures in Supplementary Fig. S2. If I_{DS} were dominated by direct tunneling, R_{DS} *vs.* V_{DS} characteristics would not depend on the temperature. In our vertical spin MOSFET, when the temperature was changed from 300 K to 3.8 K, R_{DS} was changed from 2 kΩ to 1.2 MΩ at V_{DS} = -10 mV. The strong temperature dependence of R_{DS} is experimental evidence of the indirect tunneling via defect states.

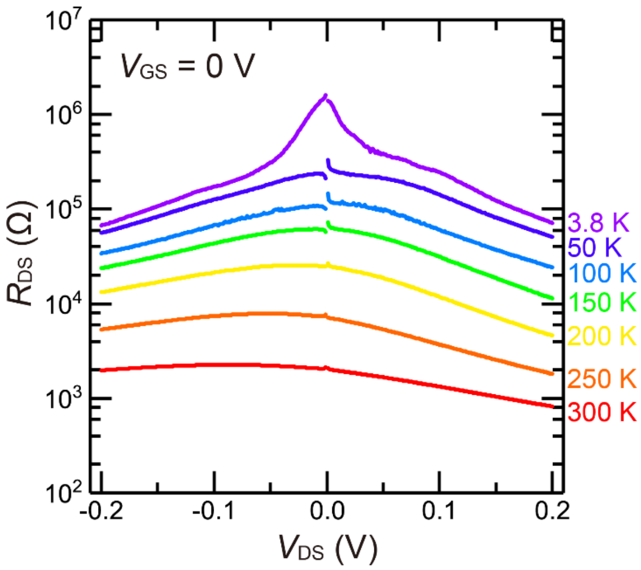

FIG. S2. Drain-source resistance R_{DS} as a function of V_{DS} at various temperatures in our vertical spin MOSFET. Here, V_{GS} = 0 V.

Supplementary Note 4. Estimation of the Curie temperatures of the top and bottom GaMnAs layers.

To estimate the Curie temperature T_C of the GaMnAs layers, we measured the temperature dependence of the major loop, as shown in Fig. S3. Here, the drain-source voltage V_{DS} is -5 mV, the gate-source voltage V_{GS} is 0 V and the external magnetic field H is applied along the [-110] direction. TMR was observed up to 20 K and disappeared at 30 K, as shown in Fig. S3. Thus, considering that the top GaMnAs layer has a higher T_C than the bottom GaMnAs layer in GaMnAs / GaAs / GaMnAs systems,[3] the T_C of the bottom GaMnAs layer is estimated to be ~30 K. To estimate the T_C of the top 10nm-thick GaMnAs layer, we prepared another heterostructure composed of $Ga_{0.94}Mn_{0.06}As$ (10 nm, the same thickness and the same Mn content) / GaAs (100 nm) on a semi-insulating (SI) GaAs (001) substrate using growth conditions similar to those for the top GaMnAs electrode of the GaMnAs/ GaAs/ GaMnAs sample described in our manuscript. We measured the temperature (T) dependence of the two-terminal resistance R of the GaMnAs layer using a patterned rectangular bar with a width of 25 μm and a length of 150 μm (the black solid curve in Fig. S4). The peak in the dR/dT – T characteristic (the red solid curve in Fig. S4) is known to correspond to the T_C in the GaMnAs film.[4] Therefore, we can estimate the T_C of the top GaMnAs layer to be ~53 K (indicated by the red arrow). Post-growth annealing was not carried out for the GaMnAs/ GaAs/ GaMnAs sample described in our manuscript.

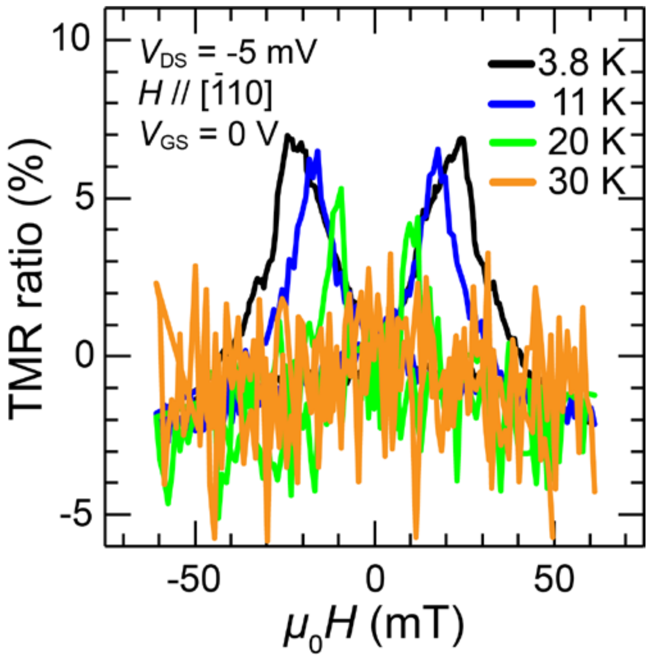

FIG. S3. TMR ratio as a function of the external in-plane magnetic field H along the [-110] direction at various temperatures. Here, the drain-source voltage V_{DS} is -5 mV and the gate-source voltage V_{GS} is 0 V.

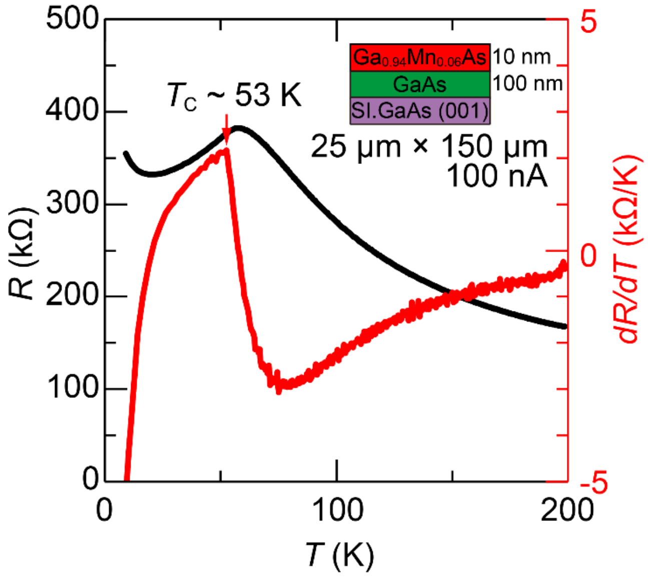

FIG. S4. Temperature T dependence of the two-terminal resistance R (the black solid curve, left axis) and the derivative of R with respect to T, dR/dT (the red solid curve, right axis). For the measurement, a patterned rectangular bar with a width of 25 μm and a length of 150 μm is used. The bias current used for the measurements is 100 nA. The red arrow indicates the T_C. The inset in Fig. S4 shows the schematic illustration of the heterostructure used for estimating the T_C of the top GaMnAs layer.

Supplementary Note 5. Top-view optical microscope photograph of a GaMnAs-based vertical spin MOSFET.

To confirm whether the GaMnAs-based vertical spin MOSFET is fabricated properly or not, we took an optical microscope photograph of the device, as shown in Fig. S5. The comb-shaped drain electrode with 20 comb teeth is formed and the gate electrode is deposited on the comb-shaped drain electrode covered with the HfO_2 layer.

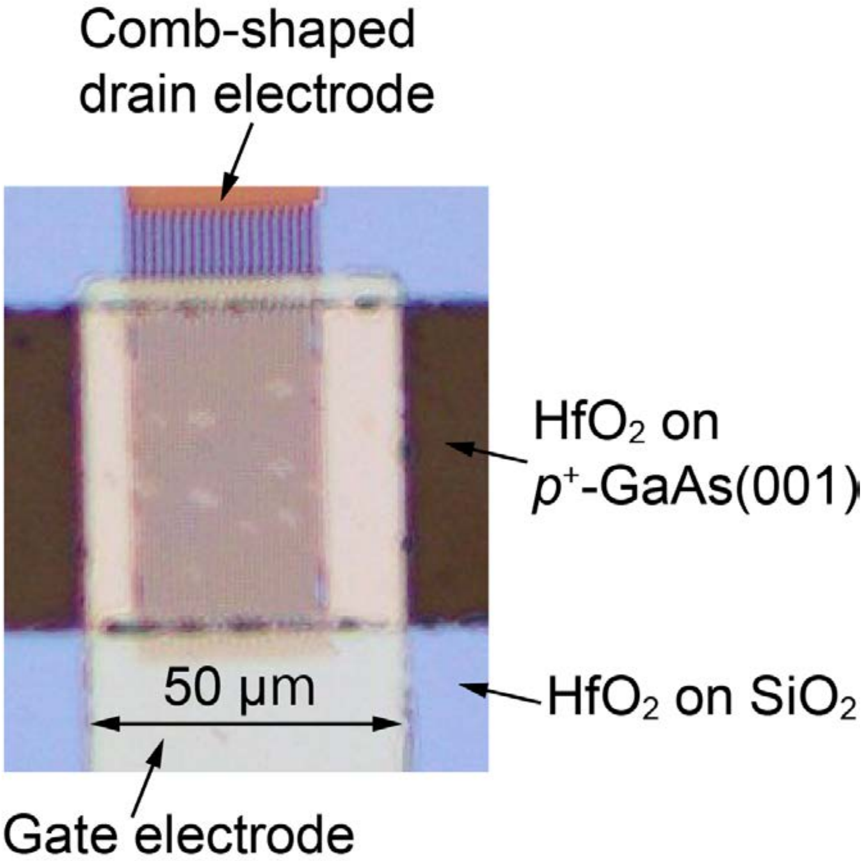

FIG. S5. Optical microscope photograph (top view) of our device.

References

S1. Ohya, S., Muneta, I., Hai, P. N. & Tanaka, M. GaMnAs-based magnetic tunnel junctions with an AlMnAs barrier. *Appl. Phys. Lett.* **95,** 242503 (2009).

S2. Ohno, Y., Arata, I., Matsukura, F. & Ohno, H. Valence band barrier at (Ga,Mn)As/GaAs interfaces. *Phys. E Low-Dimensional Syst. Nanostructures* **13,** 521–524 (2002).

S3. Chiba, D., Takamura, K., Matsukura, F. & Ohno, H. Effect of low-temperature annealing on (Ga,Mn)As trilayer structures. *Appl. Phys. Lett.* **82,** 3020–3022 (2003).

S4. Novák, V. *et al.* Curie Point Singularity in the Temperature Derivative of Resistivity in (Ga,Mn)As. *Phys. Rev. Lett.* **101,** 77201 (2008).
